# 

**Published:** 1898-04

**Authors:** 


### CONTENTS.

ORIGINAL COMMUNICATIONS. PAGE

Treatment of Pulpless Teeth with Hydrogen Dioxide, Ethereal Solution. By George S. Allan, D.D.S., New York . . . . . 201
Studies in Evolution. By Eugene S. Talbot, M.D., D.D.S., Chicago, Ill. . . . 206
The Past and Present in Dentistry. By C. N. Peirce, D.D.S., Philadelphia . . 207
The Method of using Soft Gold-Foil as practised and taught by Dr. Dunning. By Charles O. Kimball, M.D., New York . . . . . 211

ABSTRACTS AND TRANSLATIONS.

Iodoform Substitutes . . . . . 216
Liquefied Atmospheric Air . . . . . 220

REPORTS OF SOCIETY MEETINGS.

The New York Institute of Stomatology . . . . . 222
Academy of Stomatology . . . . . 230

EDITORIAL.

The Relative Value of Technical Instruction . . . . . 243
The Southern Branch of the National Dental Association . . . . . 246
The Revival of Bad Methods . . . . . 247
Liquid Air . . . . . 248

BIBLIOGRAPHY . . . . . 249

OBITUARY.

Dr. Henry S. Chase . . . . . 258
Dr. Alonzo Boice . . . . . 258

NOTES AND COMMENTS . . . . . 259

CURRENT NEWS.

Dental Society of the State of New York . . . . . 263
Pennsylvania State Board of Dental Examiners . . . . . 264
Kansas State Dental Association . . . . . 264
Illinois State Dental Society . . . . . 264
Iowa State Dental Society . . . . . 264

